# Pleomorphic Adenoma of the Submandibular Gland: A Case Report

**DOI:** 10.31729/jnma.4001

**Published:** 2019-02-28

**Authors:** Prakash Khanal

**Affiliations:** 1 Department of ENT-Head and Neck Surgery, Nepal Police Hospital, Maharajgunj, Kathmandu, Nepal

**Keywords:** *pleomorphic adenoma*, *salivary gland*, *submandibular gland tumours*

## Abstract

Salivary gland tumours are relatively rare and constitute about 3-4 % of head and neck tumours. Most of the tumours arise from parotid glands. Submandibular gland tumours are very rare. Pleomorphic adenoma of the submandibular gland is exceedingly rare tumour. Very few studies have been reported in the literature that is exclusively conducted on pleomorphic adenoma affecting submandibular gland. Patients usually present with a slow growing, painless and mobile mass without any other associated symptoms. Radiologic studies are usually unable to differentiate benign from malignant tumours in most cases. Recurrence is rare with complete en bloc excision of the tumour along with submandibular gland. Prognosis is excellent except for the rare cases of malignant transformation. This paper describes a case of pleomorphic adenoma affecting submandibular gland with brief review of current literature on submandibular gland tumours.

## INTRODUCTION

Salivary gland tumours are relatively rare and constitute 3% to 4% of head and neck tumours. About 80% of these tumours are benign. Approximately 70% of salivary tumours are located in the parotid gland.^1^ Submandibular gland tumours constitute about 10% of salivary tumours. Pleomorphic adenoma is the most common salivary gland tumour.^2^ It comprises 60-80% of parotid and 50% of submandibular gland tumours.^1–3^ This paper describes a case of Pleomorphic adenoma involving submandibular gland.

## CASE REPORT

**Figure 1.**
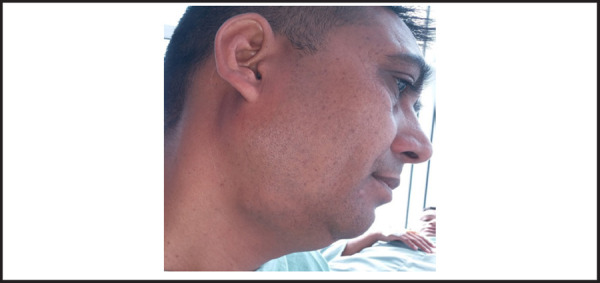
Facial profile showing swelling in the right submandibular region.

A 38-year-old male presented with history of swelling in the right submandibular region for two years. The swelling was insidious in onset, slow growing and painless. Past medical history was not significant. Neck examination revealed swelling in the right submandibular region measuring approximately 3 cmx3 cm in size and oval in shape (Figure 1). On palpation the swelling was firm, non-tender, mobile with well-defined borders and normal overlying skin. Swelling was ballotable on bidigital palpation. No hypoglossal or marginal mandibular nerve palsy or neck lymphadenopathy was present.

**Figure 2.**
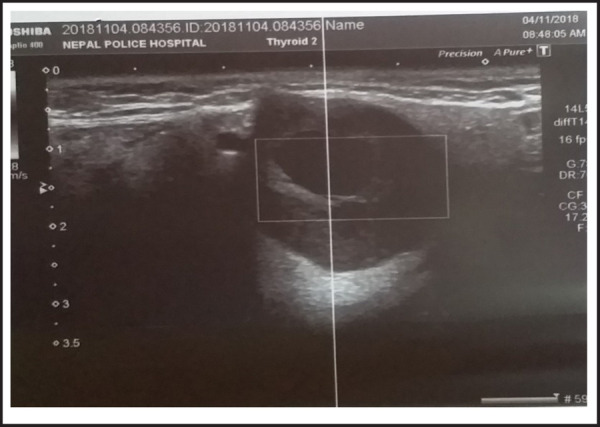
USG neck showing a 3.0 × 2.4 × 2.1 cm well defined Hypoechoic lesion in right submandibular gland with cystic component.

A provisional diagnosis of tumour of right submandibular gland was made based on history and examination findings. Patient was advised for Ultrasonogram (USG) of neck to know the type and extent of the lesion. USG neck revealed a 3.0 × 2.4 × 2.1 cm well defined complex lesion in right submandibular gland with a thin rim of normal salivary gland tissue (Figure 2). Cystic component was seen on the lesion. No abnormal vascularity or calcification was seen within the lesion. Fine Needle Aspiration Cytology (FNAC) was done which was consistent with pleomorphic adenoma.

**Figure 3.**
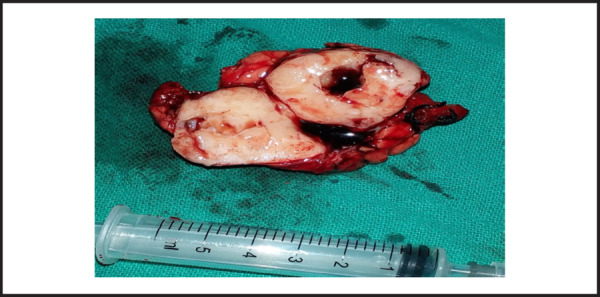
A well-encapsulated, heterogeneous hard mass with cystic area on center of mass on cut section.

**Figure 4.**
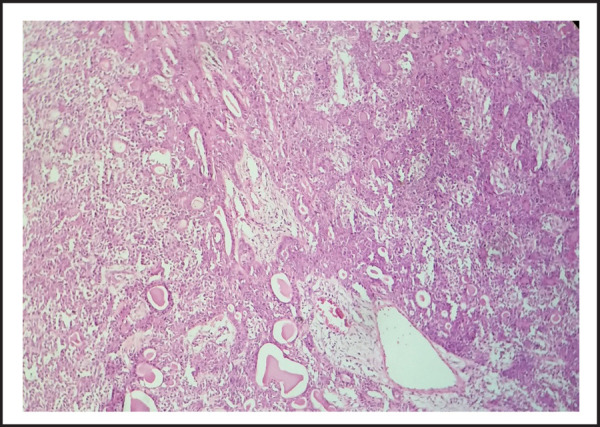
Tumour showing epithelial and mesenchymal components. H & E stain (100x)

A provisional diagnosis of pleomorphic adenoma of right submandibular gland was made and excision of the gland was done under general anesthesia. En bloc resection of tumour along with gland was done with precise dissection (Figure 3). Post-operative period was uneventful. Patient was discharged on 6^th^ postoperative day. Histopathological examination reported a well encapsulated tissue comprising of both epithelial and mesenchymal components (Figure 4). Epithelial cells were arranged in solid sheets, tubules, acini and rods. Most of the tubular structures composed of inner ductal epithelial and outer myoepithelial layer. The fibromyxoid stroma comprised of mucoid connective tissue with areas of cartilaginous tissue. Normal looking salivary gland tissue present in the periphery of lesion. No features of dysplasia or malignancy were seen. The final diagnosis of Pleomorphic Adenoma of right submandibular gland was made.

## DISCUSSION

Salivary gland tumours are relatively rare and constitute 3% to 4% of all head and neck tumours and most commonly affect parotid gland.^1^ Pleomorphic adenoma is by far the most common salivary gland tumour.^2^ Submandibular gland tumours are much less common than parotid tumours and constitute about 10% of salivary tumours. Pleomorphic adenoma is also the most frequent benign tumour arising in submandibular gland and constitutes about 50% of submandibular tumour.^4^ Adenoid cystic carcinoma is the most common malignancy of submandibular gland. Others includes Carcinoma ex pleomorphic adenoma, mucoepidermoid carcinoma, non-Hodgkin's lymphoma and squamous cell carcinoma.^5^

Pleomorphic adenoma of the submandibular gland is relatively uncommon. It occurs in patients of all ages, with the highest incidence reported in the fourth to fifth decades. Both sexes are affected equally. It usually presents as a painless, slow-growing, firm, mobile mass in the submandibular triangle without fixation to the floor of the mouth or the mandible. Neck lymphadenopathy and nerve compromise are usually absent.^6^ The cut surface is characteristically solid and may be hard, rubbery, or soft in consistency with a whitish gray to pale yellow color. Pleomorphic adenomas of the major salivary glands have a capsule varying in thickness. Pleomorphic adenomas show a remarkable histologic diversity and are also called benign mixed tumours. These benign tumours arise from a mixture of ductal and myoepithelial cells and show both epithelial and mesenchymal differentiation. Epithelial components are present in the form of ducts, nests, cords, or solid sheets of cells and myoepithelial cells in fibrocollagenous, myxochondroid, or chondroid background.

Ultrasonography can differentiate solid from cystic lesions in the salivary glands. It is also useful in guiding a biopsy (both FNAC and core biopsies). USG can diagnose most of the salivary gland diseases.^7^ Pleomorphic adenomas are typically hypoechoic and may show posterior acoustic enhancement. Most salivary gland tumours are investigated by FNAC. The accuracy for the diagnosis of benign or malignant salivary gland tumour by FNAC is around 80-90%.^8^ FNAC also helps to differentiate between tumours and inflammatory conditions or enlarged lymph nodes. Computerized Tomography (CT) scan or Magnetic Resonance Imaging (MRI) are the gold standard radiological tools for lesion arising from the salivary glands. The final pathologic diagnosis is always established based on the histopathological findings after surgical excision.

En bloc resection of the tumour and the involved gland, is the treatment of choice for pleomorphic adenoma of the submandibular gland.^9^ Enucleation alone carries a high risk of recurrence due to pseudopod-like extensions of tumour. Injury to the marginal mandibular nerve is the most common complication leading to temporary or permanent paralysis due to the stretching or compression of the nerve.^10^ Malignant transformation of pleomorphic adenoma is rare and occurs most frequently in patients with long-standing tumour. The risk of malignant transformation in pleomorphic adenoma is 1.5% within the first 5 years of diagnosis but increases to 10% if observed for more than 15 years.^11^ Therefore, early definitive treatment is strongly recommended.

## Consent

**JNMA Case Report Consent Form** was signed by the patient and the original is attached with the patient's chart.

## Conflict of Interest

**None.**
